# Cytotoxic and Membrane Cholesterol Effects of Ultraviolet Irradiation and Zinc Oxide Nanoparticles on Chinese Hamster Ovary Cells

**DOI:** 10.3390/molecules23112979

**Published:** 2018-11-15

**Authors:** Regina E. Kuebodeaux, Paul Bernazzani, Thi Thuy Minh Nguyen

**Affiliations:** Department of Chemistry and Biochemistry, Lamar University, Beaumont, TX 77710, USA; rekuebodeaux@gmail.com (R.E.K.); paul.bernazzani@lamar.edu (P.B.)

**Keywords:** UV-radiation, ZnO, toxicity, sterol content, cholesterol

## Abstract

Zinc Oxide (ZnO) nanoparticles are suspected to produce toxic effects toward mammalian cells; however, discrepancies in the extent of this effect have been reported between different cell lines. Simultaneously, high levels of ultraviolet (UV-C) radiation can have carcinogenic effects. The mechanism of this effect is also not well understood. Due to similarities in phenotype morphology after cell exposure to ZnO nanoparticles and UV-C irradiation, we emit the hypothesis that the toxicity of both these factors is related to damage of cellular membranes and affect their sterol content. Wild-type Chinese Hamster Ovary (CHO-K1) cells were exposed to ZnO nanoparticles or UV-C radiation. The amount of absorbed ZnO was determined by UV-visible spectroscopy and the changes in sterol profiles were evaluated by gas chromatography. Cell viability after both treatments was determined by microscopy. Comparing morphology results suggested similarities in toxicology events induced by ZnO nanoparticles and UV exposure. UV-C exposure for 360 min disrupts the sterol metabolic pathway by increasing the concentration of cholesterol by 21.6-fold. This increase in cholesterol production supports the hypothesis that UV irradiation has direct consequences in initiating sterol modifications in the cell membrane.

## 1. Introduction

Advances in the field of nanotechnology where nanoparticles are commonly used in catalysts, sensors, and photovoltaic devices [1,2,3], as well as in the biomedical field for nanovaccines, nanodrugs, and diagnostic imaging tools [4,5,6,7], may also be associated with different health issues. The potential health impact nanoparticles raise concerns the fact that they have been incorporated into products used on a daily basis, as well as in medical products. There are several factors that determine nanoparticle cytotoxicity: the size, shape, surface charge, hydrophobicity, and heavy metal content [8]. It is important to understand what happens on a cellular level after nanoparticles have entered the human body through inhalation, ingestion, or absorption through the skin. Typically, the smaller the nanoparticle, the more cytotoxic it becomes due to its high surface area to volume ratio. Some studies revealed that not only can nanoparticles pass through cellular membranes, but they can also pass through the blood-brain barrier, which can result in organ deposition and interaction with biological systems and, therefore, alter the chemical biosynthesis [7,9,10,11].

Zinc Oxide (ZnO) nanoparticles are widely used [12] and recent investigations discuss their cytotoxicity, in particular, the relation between Zn^2+^ cations and the generation of reactive oxidative species, which contribute significantly to the degenerescence of macromolecules and, therefore, to the toxicity of Zn^2+^ [13,14]. In addition, the interaction of zinc in mammalian cells is complex. For example, zinc has been found to bind to enzymes related to sterol regulation [15] and help regulate the structure of cell membranes [16]. Hence, the possibility exists that ZnO nanoparticles will affect the sterol biosynthetic pathway, impacting the stability of the membrane and the phagocytosis process.

Another process that may affect sterol production in cells is the exposure to ultraviolet (UV) radiation. UV radiation is categorized into three wavelength ranges: long wave UV-A (315–400 nm), medium wave UV-B (280–315 nm), and short-wave UV-C (100–280 nm) [17,18,19,20]. The most energetic region, UV-C, is typically shielded by the ozone layer of the earth’s atmosphere; however, due to ozone damage in recent years, the possibility exists that these radiations may reach the surface of the earth and affect sterol production in microalgae [21]. The effects of UV irradiation on mammalian cells remains unclear. In addition, UV radiation causes many harmful effects particularly regarding skin malignancies and severe damage to nucleic acids leading to DNA mutations; however, it can also be beneficial by creating vitamin D3 and is coupled in drug therapy applications for curing skin diseases like psoriasis and vitiligo [17,21]. The high energy radiation can cause damage to living organisms either by direct absorption through cellular chromophores or by indirect means through photo-sensitization mechanisms [21]. The indirect mechanism results in the formation of a reactive oxidative species, which can damage DNA, proteins, fatty acids, and saccharides [2]. There are therefore parallels between the effect of exposure to UV-C radiation and zinc ions. Both seem to affect the sterol biosynthetic pathways, and both can cause the generation of reactive oxidative species that lead to harmful effects.

We report the comparison of the cytotoxic effect of ZnO nanoparticles and UV-C irradiation on Chinese Hamster Ovary (CHO-K1) cells. We postulate that when exposed to either ZnO nanoparticles or UV radiation, the stability of cellular membranes will be modified because of variations in the sterol profile. Several analyses were performed on CHO cells after exposure to ZnO nanoparticles and UV-C irradiation to determine the amount of ZnO nanoparticles that cells can absorb, and alterations in cell growth and viability, DNA, and sterol profiles. The amount of ZnO nanoparticles was quantified using UV-Visible spectroscopy. Cell morphology and viability was determined with the use of compound light microscopy and the trypan blue exclusion method. Changes in DNA composition were visualized using agarose gel electrophoresis and sterol profile modifications were analyzed by gas chromatography.

## 2. Results and Discussion

### 2.1. UV-Visible Spectroscopy after ZnO Treatment

The ZnO nanoparticles used in this study were less than 100 nm in size. This is on the same scale as some biomolecules; therefore, we hypothesize that they are capable of passing through the protective cellular membrane. If so, nanoparticles could potentially enter the interior environment of the cell and this effect needs to be quantified.

**Figure 1:**
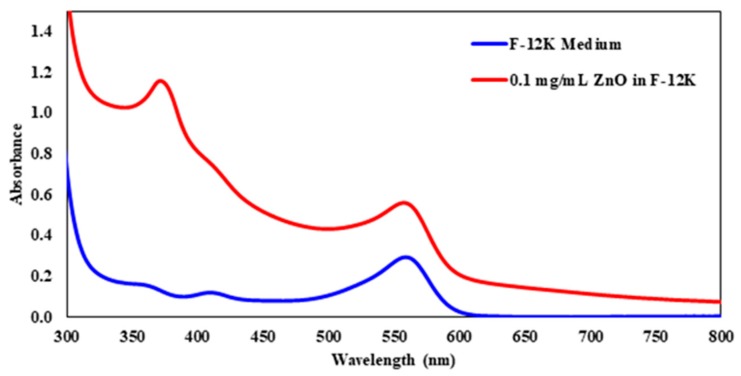
The UV-Visible Spectra of the controls: Ham’s F-12K medium and 0.1 mg/mL of ZnO solution in an F-12K medium.

UV-visible spectroscopy was used to determine the amount of ZnO that CHO-K1 cells absorbed after 24 h of exposure, by first determining the wavelength corresponding to the maximum absorbance (λmax) of the ZnO nanoparticles dissolved in the F-12K medium. Figure 1 displays the UV-visible spectra of the controls used: Ham’s F-12K and 0.1 mg/mL ZnO in the F-12K medium. Nanoparticles tend to scatter the light when they are analyzed by UV-Visible spectroscopy, which shifted the baseline upward. The baseline shift was taken into account when determining the absorbance at λmax. The λmax of the 0.1 mg/mL ZnO solution was determined to be at 372 nm. The absorbance at the wavelength was calculated by subtracting the absorbance at 390 nm from the absorbance at the highest peak. Ham’s F-12K was also analyzed and its λmax was found to be around 550 nm.

**Figure 2:**
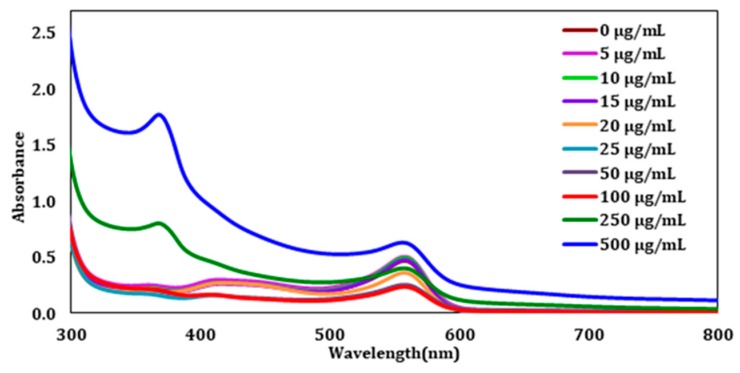
The UV-Visible Spectra of the supernatant of wash 1 after treatment with different ZnO concentrations.

After establishing the λmax from the control sample, each ZnO exposed sample was analyzed by UV-Vis spectroscopy. This was performed to understand how the CHO cells respond to different ZnO concentration doses, and to determine at what concentration the cells reach saturation. Once the ZnO treatment was complete, the cells were pelleted by centrifugation and the clear supernatant liquid was analyzed to determine the number of unabsorbed ZnO nanoparticles. The UV-visible spectra after this first wash can be seen in Figure 2. The figure displays the unabsorbed ZnO absorbances from the wavelength range of 300–800 nm. From the lower concentrations (0–100 µg/mL), it is challenging to see a difference with the addition of ZnO. However, at 250 and 500 µg/mL, an absorbance at 372 nm can be observed clearly.

**Figure 3:**
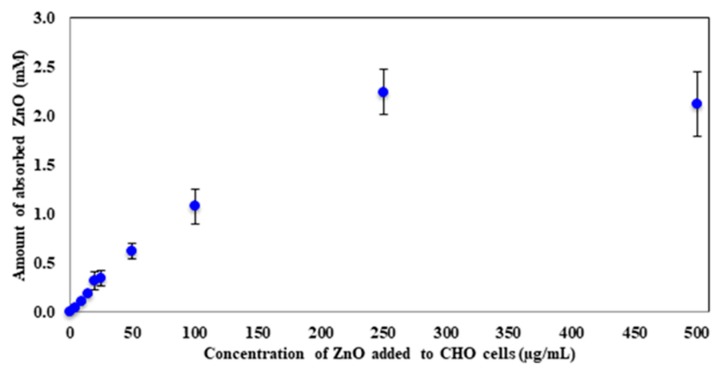
The average amount of absorbed ZnO (mM) after 24 h of ZnO treatment. Error bars represent the standard deviation of triplicates.

The absorbance at 372 nm was used to determine the concentration of ZnO that CHO-K1 cells absorbed for each of the samples with the help of Equation (1).

(1) $$C_{s}=C_{Initial\;\left[ZnO\right]}-\left[\frac{\left(\;C_{c}\;\times \;A_{s}\right)}{A_{c}}\right]$$

where *C_s_* is the concentration of the sample and *A_s_* is the absorbance at 372 nm for the sample. *C_c_* and *A_c_* correspond to the concentration and absorbance at 372 nm for the ZnO control. *C_Initial [ZnO]_* is the starting concentration of ZnO that each sample received. The concentration of each sample was calculated in mM and was plotted against the concentration of ZnO that was initially added to CHO cells (Figure 3). A steady increase in the amount of absorbed ZnO with an increase in dosage was observed. As expected, the absorption reaches a plateau when large concentrations of ZnO are present, indicating the possibility that the at least 250 µg/mL ZnO cause CHO-K1 cells to become saturated and no longer absorb or uptake the nanoparticles. The maximum amount these mammalian cells absorbed was 2.2 ± 0.2 mM ZnO. The 250 µg/mL sample had an initial concentration of 3.1 mM; therefore, around 70% of the ZnO nanoparticles were absorbed by the cells at this concentration. For the 500 µg/mL sample, 6.2 mM was the starting concentration of ZnO, which corresponds to around 35% absorption.

The results indicate that CHO-K1 cells are indeed capable of absorbing ZnO nanoparticles after 24 h of exposure. The amount absorbed by the cell increased with increasing ZnO dose and a saturation parameter was established at 250 µg/mL. To determine the toxicity of these nanoparticles, cell morphological changes and viability were examined by microscopy.

### 2.2. Cell Viability and Morphology

Cell viability and cell morphology help establish the degree of toxicity of external agents such as the addition of ZnO nanoparticles or UV-C irradiation. CHO-K1 cell viability and morphology were determined using a BioExpress GeneMate inverted microscope and trypan blue exclusion test. Trypan blue is a staining technique that allows for the observer to differentiate which cells are no longer viable. The unhealthy/dead cells have damaged cell membranes. This allows the cell to absorb the trypan blue dye and become blue, while the healthy, viable cells are not stained. Once the viable cells can be identified, the percent viability and viable cell concentration (viable cells/mL) can be calculated using a hemocytometer and Equations (2) and (3).

% Viability = [1 − (Number of blue cells ÷ Number of total cells)] × 100(2)

Viable cells/mL = Average number of viable cells × 16 × 104(3)

**Figure 4:**
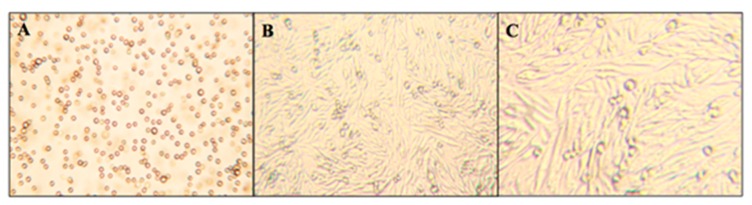
The wild-type CHO cell micrographs of the Initial Suspension Stage at 250× (**A**) and the Mature Adhered Phase at 250× (**B**) and at 400× (**C**).

CHO-K1 cells are adherent epithelial cells, meaning that when the cell culture matures, the cells attach to the culture flask. After the cells have adhered to the culture flask, their morphology changes to become more stretched and oblong, whereas the younger cells that have not adhered have a circular morphology. Figure 4 displays micrographs of these two different growth stages that CHO-K1 cells exhibit: the initial suspension stage (panel A) and the mature adherent stage (panels B and C). While cells in the suspension stage have a rounded morphology, mature cells in the adherent stage show significant elongation. The rounded cells still observed in panels B and C are expected and represent newly formed immature cells.

### 2.3. Cell Viability and Morphology after ZnO Treatment

**Figure 5:**
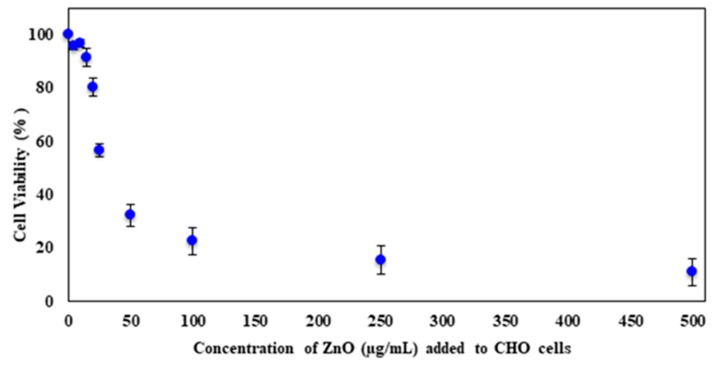
The average percent cell viability after 24 h of incubation with ZnO. Error bars represent the standard deviation of triplicates.

Figure 5 shows the percent viability curve from 0–500 µg/mL ZnO after cell incubation in the presence of different amounts of nanoparticle for 24 h at 37 °C with 5% CO_2_. The cells were grown in the presence of ZnO in a complete growth medium containing Ham’s F-12K media with 10% FBS and antibiotics. Cell viability after ZnO exposure was characterized with a decrease in the percent viability as the concentration of ZnO increased. From the concentrations of 15 µg/mL and lower, there was not much influence on viability. At 15 µg/mL ZnO, 91.6% of the cells were still viable. As the amount is increased to 20 µg/mL ZnO, it fell to 80.3%. A drastic drop of almost 24% in viability was observed between the ZnO concentrations of 20 to 25 µg/mL. A similar decrease in percentage was found from 25 to 50 µg/mL ZnO. Higher amounts resulted in a gradual decline in viability and, eventually, the lowest viability of 11.0% was observed at the highest concentration of 500 µg/mL ZnO.

**Figure 6:**
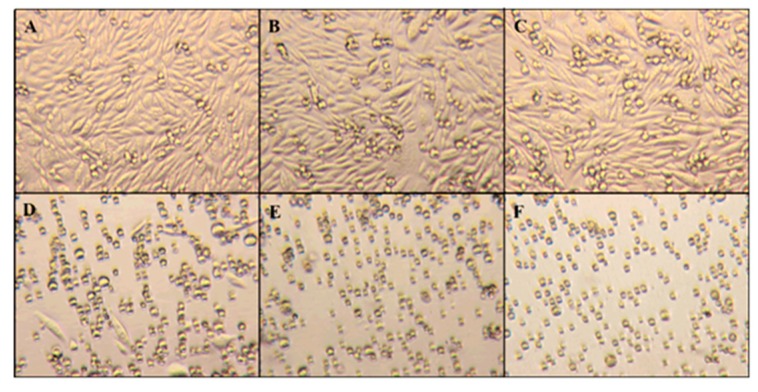
The CHO cell morphology micrographs (400×) of 0 µg/mL control (**A**), 10 µg/mL (**B**), 15 µg/mL (**C**), 25 µg/mL (**D**), 50 µg/mL (**E**), and 100 µg/mL ZnO (**F**) after 24 h of exposure.

As expected, viability analysis confirmed that ZnO nanoparticles are toxic to CHO-K1 cells at higher concentrations, but morphology changes provide additional information about the toxicity of these nanoparticles. Changes in morphology could mean that the natural behavior of the cell is altered. Figure 6 shows the microscopy images of the culture flasks using an inverted microscope. The different panels represent typical behavior under different ZnO amounts. Images indicate that the morphology of CHO-K1 cells was directly impacted as the concentration of ZnO present increased. At concentrations of 20 µg/mL ZnO and below, the cells were able to maintain their natural adherent behavior. At 25 µg/mL of ZnO, some CHO cells were able to maintain the adherent morphology, but the majority of the cell culture expressed a round physiology. However, at amounts exceeding 25 µg/mL ZnO, the CHO-K1 cells stayed in the media and grew similar to suspension cells and stayed circular in form.

The presence of Zinc oxide nanoparticles not only impacts the viability of CHO cells, but also influences their physical characteristics. When 25 µg/mL of ZnO and above were introduced, the cells were no longer able to completely adhere to the culture flask. This indicates that the mode of toxicity caused the natural function of the cell to change. These results were compared with the viability and morphological deviations after UV-C exposure.

### 2.4. Cell Viability and Morphology after UV-C Exposure

**Figure 7:**
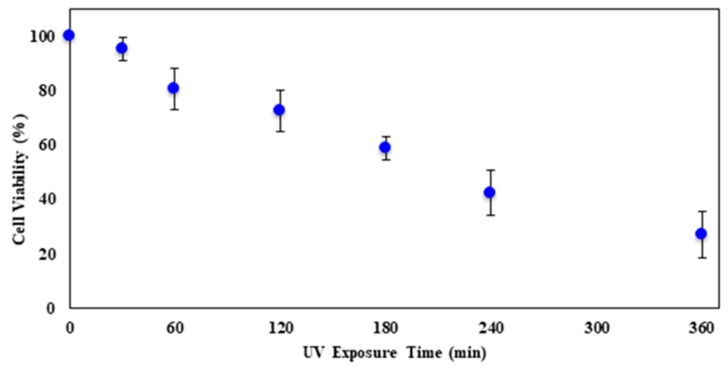
The average percent cell viability after different UV-C exposure times. Error bars represent the standard deviation of triplicates.

Figure 7 shows the viability curve of CHO cells as they were exposed to UV-C for longer times. With the increase in UV exposure time, there was a decrease in cell viability. The result provides for an almost linear relation, rather than an exponential decrease as the ZnO treatment provided. On average, cell viability dropped by 12.1%/min during the span of 360 min of UV exposure. At the maximum UV exposure time of 360 min, the number of viable cells was 27.0%.

**Figure 8:**
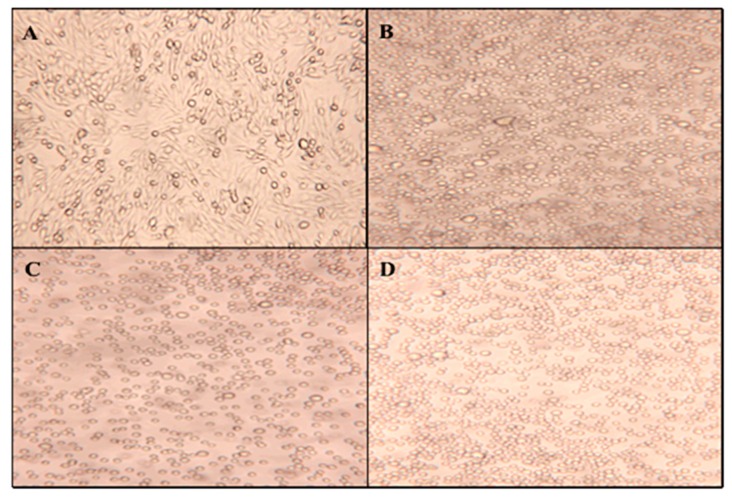
The CHO cell morphology micrographs (250×) of Wild-type CHO-K1 (**A**), 60 min (**B**), 180 min (**C**), and 360 min (**D**) of UV exposure.

To determine the effect of UV-C irradiation on cell morphology, Figure 8 displays micrographs taken directly from the petri dish after UV exposure times of 0, 60, 180, and 360 min. All exposure times resulted in suspension-like cell cultures with the cellular structure staying in circular form. UV-C irradiation showed a similar, although stronger, impact on CHO cell morphology compared to exposure to ZnO nanoparticles. These results indicate that UV-C exposure has an extreme impact on cell morphology, but do not reveal how this radiation affects the cell membrane. As previously discussed, because the phenotype shows that cell membranes are affected, the toxicity can be attributed to either the formation of reactive oxidative species or an effect on the sterol biosynthetic pathway, which directly impacts the membrane. This latter assumption can be evaluated through gas chromatography analysis of the extracted membrane sterols after each UV exposure time.

Evaluation of the possible damage caused by reactive oxidative species on DNA can also be determined. Alterations in the DNA were first assessed before analyzing the sterol profile.

### 2.5. Agarose Gel Electrophoresis after ZnO Treatment

**Figure 9:**
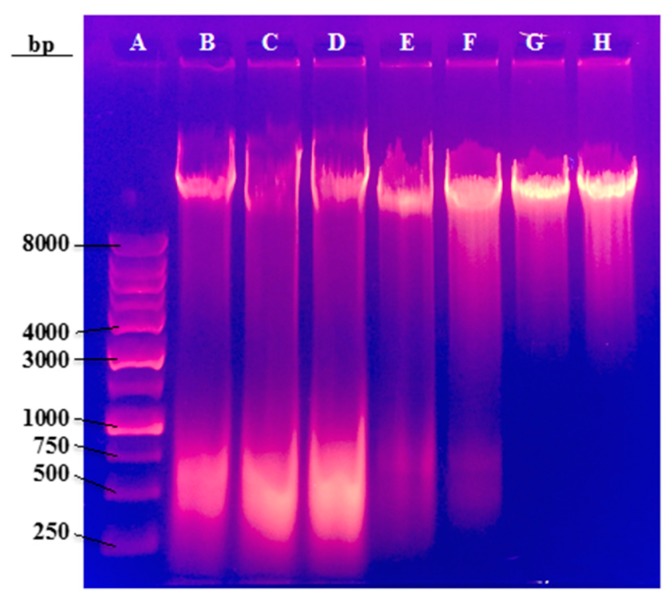
A total of 1% of Agarose gel of the samples exposed to different concentrations of ZnO: DNA Ladder (**A**), 0 µg/mL (**B**), 5 µg/mL (**C**), 10 µg/mL (**D**), 15 µg/mL (**E**), 25 µg/mL (**F**), 50 µg/mL (**G**), and 100 µg/mL (**H**).

Agarose gel electrophoresis was used to evaluate possible alterations in CHO-K1 genomic DNA after incubation of different concentrations of ZnO. Electrophoresis was performed on whole genomic DNA without the use of restriction enzymes. Since restriction enzymes were not used, distinct bands were not observed in the agarose gel. Figure 9 displays the electrophoresis gel following this experiment.

The samples of 0 to 15 µg/mL ZnO (Lanes B–D) have a prominent band smear towards low molecular weight around 500 base pairs. This band corresponds to RNA molecules that were also purified during DNA extraction. Lanes E and F (20 and 25 µg/mL ZnO) displayed the bands as well, but were in trace amounts that resulted in faint bands. The higher concentrations of 50 and 100 µg/mL of ZnO (lanes G and H) had no appearance of the RNA band. This indicates that with increased ZnO dosage, CHO-K1 cell’s RNA is destroyed. If RNA is no longer synthesized within the cell, then DNA replication cannot occur as well. When DNA replication is halted, the cell can no longer produce the necessary proteins for maintenance, survival, and the creation of a healthy internal environment. This explains why ZnO has such a direct effect on cell viability and morphology at the higher concentration doses.

Termination of cell replication is an extreme consequence of toxicity brought on by ZnO nanoparticles; therefore, membrane sterol modifications were not investigated. However, since the UV exposed CHO samples did not present this effect, modifications in their sterol profile was explored.

### 2.6. Gas Chromatography after UV Exposure

**Figure 10:**
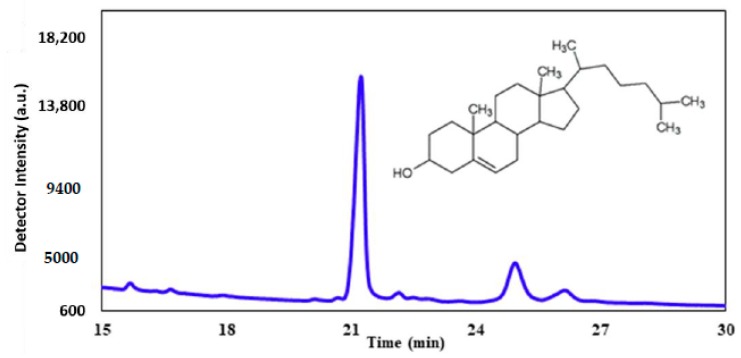
The GC chromatogram of standard cholesterol (1.0 mg/mL).

We emit the hypothesis that exposure to UV-C results in a direct effect on cell membrane integrity. Gas chromatography was utilized to analyze the sterol profile of CHO-K1 cells after UV-C exposure. Cholesterol was used as a standard at a concentration of 1.0 mg/mL. This specific sterol was chosen as a standard because cholesterol is the most abundant sterol found within mammalian cellular membranes. Figure 10 represents the typical GC trace of the cholesterol standard. The prominent peak at around 21 min corresponds to pure cholesterol, while the smaller peaks at around 25 and 26 min represent derivatives.

**Figure 11:**
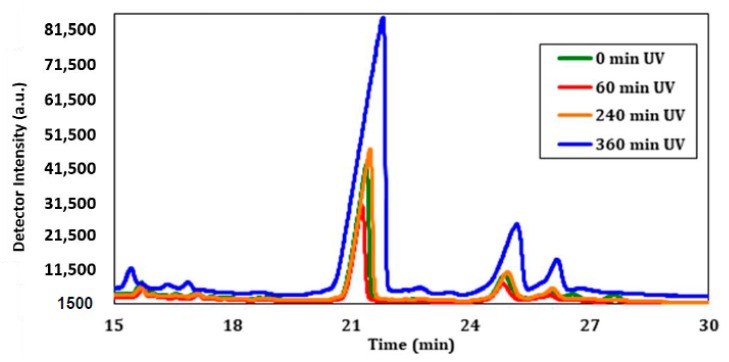
The GC chromatograms after CHO UV exposure for 0, 60, 240, and 360 min.

The sterol profiles after 0, 60, 240, and 360 min of UV-C exposure showed interesting results related to the deviation of cholesterol content. The GC chromatograms in Figure 11 show the peak related to cholesterol at a retention time of about 21 min, but do not take into account the number of cells that are viable in each sample. A decrease was observed in the amount of cholesterol after 60 min of UV exposure when compared to the sample that was not exposed. However, after 240 min of exposure, there was a slight increase in the amount of cholesterol that would generally be present under normal conditions. Intriguingly, after 360 min of exposure, the amount of cholesterol was substantially greater compared to all other samples. This provides evidence that UV-C irradiation has a direct impact on cholesterol found within the cell membrane of CHO-K1 cells, particularly after longer exposures.

Direct evaluation of the GC peak area does not demonstrate the true impact of cholesterol modification after UV exposure because each sample had a different concentration of cells that were left viable. Table 1 displays the calculated concentrations of cholesterol before and after the viability percentage was taken into account.

**Figure 12:**
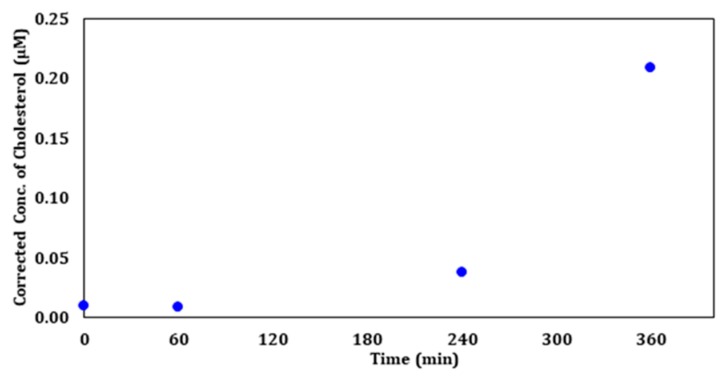
The corrected concentration of cholesterol (µM) after UV Exposure for 0, 60, 240, and 360 min.

Figure 12 displays the calculated corrected concentration of cholesterol in µM based on the viability of each exposure time. An increase in the amount of cholesterol with increasing exposure time was found when correcting for concentration based on the number of viable cells after each exposure. These results support the hypothesis that UV-C irradiation affects cell membrane cholesterol levels.

The fact that viability and cholesterol are inversely proportional implies that the increase in cholesterol undermines the integrity of the cellular membrane, which results in the change in morphology and apoptosis. DNA and protein analyses need to be performed in future studies to better understand the mechanism that stimulates the spike in cholesterol production upon UV-C exposure.

## 3. Materials and Methods

### 3.1. Cell Culture

Wild-type Chinese Hamster Ovary, CCL-61, (CHO-K1) purchased from American Type Culture Collection (ATCC, Manassas, VA, USA), were cultured in Ham’s F-12K with L-Glutamine medium (ATCC, 30-2004) containing 10% fetal bovine serum (FBS) (ATCC, 30-2020) and 1% Penicillin/Streptomycin (ATCC, 30-2300). All culture media, serum, and antibiotics were purchased from ATCC. The cells were cultured at 37 °C in an atmosphere containing 5% carbon dioxide (CO_2_) in a Steri-Cycle CO_2_ Incubator (Thermo Scientific, Waltham, MA, USA, Model 370 Series). The cultures were grown in T-25 culture flasks until 80–90% confluency before ZnO (Sigma Aldrich, St-Louis, MO, USA, number 721077) or UV-C exposure.

### 3.2. Cryopreservation of CHO-K1 Cells

Cell culture was grown until 90% confluency and checked for cell viability and contamination immediately before cryopreservation. For CHO-K1 cells, the cryopreservation medium was prepared with Ham’s F-12K with 20% FBS supplemented with 5% DMSO (ATCC) and 1% Penicillin/Streptomycin. Care was given to not add undiluted DMSO to the cell suspension as the dissolution of DMSO in aqueous solutions gives off heat.

The cells were collected by gentle centrifugation with a Thermo Scientific Sorvall Legend X1R centrifuge at 2500 rpm for 10 min, followed by the suspension of the pellet with the cryopreservation medium at a concentration of 1 × 10^6^ to 5 × 10^6^ viable cells/mL. The cryopreservation vials were labeled and 1 mL of the cell suspension was added to each vial and then sealed. The cells were allowed to equilibrate in the freeze medium at room temperature for a minimum of 15 min, but no longer than 40. This time was usually taken up in dispensing aliquots of the cell suspension into the vials. If left for longer than 40 min at room temperature, the cell viability may decline due to the DMSO. The vials were then placed on ice and stored in an −80 °C freezer for at least 24 h. Then the vials were transferred quickly to an ABS1 CryoMax Liquid Nitrogen Dewar (American BioTech Supply, Phenix Research Products, Candler, NC, USA) for long-term storage in the liquid nitrogen vapor phase. The location of vials within the Dewar was then recorded for future reference.

### 3.3. Subculturing CHO-K1 Cells

CHO-K1 cells were sub-cultured after they were 90% confluent in a T-25 flask. The culture medium was removed and discarded. Then the cell layer was rinsed with Ham’s F-12K without serum several times and discarded. This was done to remove all traces of the serum, which contains the trypsin inhibitor. After rinsing, 2 mL of 0.25% (*w*/*v*) Trypsin-0.53 mM EDTA solution (ATCC) was added to each flask. Trypsin is an enzyme that allows the adherent cells to detach from the culture flask. To facilitate dispersal, the cells were placed at 37 °C and took about 10–15 min to complete. The cells should be monitored under an inverted microscope until the cell layer is dispersed in the solution. After the cells had completely detached from the flask, F-12K media without serum was added and the cells were aspirated by gently pipetting. Cell count and viability were determined, followed by centrifugation at 2500 rpm for 5 min. The F-12K media was discarded and fresh media was added and the centrifugation process was repeated. Then complete growth medium of F-12 K supplemented with 10% FBS with 1% Penicillin/Streptomycin was added to the cell pellet and appropriate aliquots of the cell suspension were added to new T-25 culture vessels. The cultures were then incubated at 37 °C and were examined the following days to ensure cell reattachment and active growth.

### 3.4. ZnO Nanoparticle Treatment and Analysis

CHO-K1 cells were exposed to 0, 5, 10, 15, 20, 25, 50, 100, 250, and 500 µg/mL ZnO for 24 h at 37 °C with 5% CO_2_ in either 12 well-culture plates or T-25 culture vessels. The dose selection was obtained experimentally. Systematic trials over a large range of dosage showed a range where cells were still viable. Sigma Aldrich (Sigma Aldrich, St-Louis, MO, USA) provided ZnO nanoparticles that were less than 100 nm in size. Prior to ZnO incubation, the cells were cultured until 90% confluency and then were sub-cultured and split into additional flasks and grown until they were 90% confluent. Table 2 and Table 3 provides the specific volumes and concentrations used for the 24 h ZnO treatment when a 12-well plate or a T-25 flask was used, respectively.

A Thermo Scientific Evolution 260 Bio UV-Visible Spectrophotometer was utilized to determine the concentration of ZnO nanoparticles that were unabsorbed after treatment. This was done by measuring the absorbance of three supernatants from 200–800 nm of each sample. After ZnO treatment, the cells were centrifuged at 2500 rpm for 5 min and the supernatant after centrifugation was analyzed by UV-Vis spectroscopy and classified as wash 1. Then PBS was added to the pelleted cells, resuspended, and centrifuged. This supernatant was analyzed and termed wash 2. The process was repeated one more time with PBS to yield the final wash 3. The controls used were 1 mg/mL and 0.1 mg/mL ZnO in F-12K media with 10% FBS, PBS, and F-12K media with 10% FBS. Water was used as a blank to calibrate the spectrophotometer.

### 3.5. DNA Purification and Agarose Gel Electrophoresis

Chinese Hamster Ovary cell DNA was extracted and purified after ZnO treatment for 24 h at 37 °C with PureLink Genomic DNA Mini Kit provided by Invitrogen. The procedure of preparing cell lysates, purifying, and extracting DNA was performed as the kit described for mammalian cells.

Agarose cell electrophoresis was performed with 1% agarose gel, 1× Tris/Acetic Acid/EDTA (TAE) buffer (Bio-Rad, Hercules, Berkeley, CA, USA), and ethidium bromide in a Bio-Rad Mini-Sub Cell GT apparatus. A PowerPac basic power supply from Bio-Rad was used to provide the voltage current during electrophoresis. The DNA samples were prepared with 6× loading buffer to yield a 1× sample and a DNA marker was loaded during each run as a control. The agarose gel was run at 100 V for approximately 30 min and then observed under UV for analysis.

### 3.6. UV-C Irradiation Treatment

UV irradiation treatment of 60 mm Petri dishes, prepared for sterol extraction following different exposure times: 0, 30, 60, 120, 180, 240, 360 min was performed in a Luzchem (Gloucester, ON, Canada) LZC-4× photoreactor (4 UV-C lamps, dose of 60,000 mW/m^2^ with emission 235–280 nm, and peak emission at 254 nm) such that the lamps were above the samples. Prior to the addition in a petri dish, the CHO-K1 cells were cultured, sub-cultured, and then cultured until 90% confluency at 37 °C with 5% CO_2_ in F-25K culture flasks. Then the cells were added to a petri dish at a concentration of 1 × 10^6^ cells/mL with a final volume of 8 mL of Ham’s F-12K supplemented with 10% FBS media with 1% penicillin-streptomycin. After each exposure time, the cell viability was analyzed using a 0.4% solution of Trypan Blue (American BioInnovations, Sparks, MD, USA).

### 3.7. Sterol Extraction after UV-C Exposure

The cell culture remaining after the allotted UV-C exposure time was washed twice with distilled water (dH_2_O) and centrifuged at 2500 rpm for 10 min. Between each centrifugation, the clear supernatant fluid was discarded and the pellet was resuspended with fresh dH_2_O. After the washes were complete, the cell pellet was stored at −20 °C overnight and sterols were extracted the following day.

To begin the sterol extraction process, 500 µL of dH_2_O and 1 mL of 10% KOH in methanol was added to the cell pellet in a 15-mL centrifuge tube. The dH_2_O/KOH/cell solution was then transferred to a small glass test tube and 100 µL of DMSO and a boiling chip was added. The contents within the test tube were then heated to 90–100 °C and allowed to boil for about 20 min. This was followed by the addition of 500 µL of dH_2_O and then the solution was allowed to cool down to room temperature. This process allows for saponification to occur. The sterols within the cells are released because saponification causes hydrolysis of sterol esters and destroys the cellular tissues.

The free sterols were then extracted with 2 mL of hexane three times. The sterols travel to the clear organic hexane layer, and the polar compounds, such as phospholipids are attracted to the aqueous layer. Between each hexane addition, the test tubes were vortexed and centrifuged at 3300 rpm for a minute to allow the sterols to reach the organic layer more efficiently. The top organic layer was extracted and transferred to a smaller test tube and dried completely with N_2_(g) at 60 °C.

Once the hexane was completely evaporated, acetone was added to the glass tube to dissolve any residue that might be present. The sample was then vortexed, sonicated to remove sterols from the side of the glass, and dried completely with N_2_(g) at 60 °C. The same process of vortexing, sonicating, and drying with N_2_(g) was performed with methanol two times after the acetone evaporated. Once completely dry, the sterol samples are ready for GC injection after the addition of 20.0 µL of methanol. The sterol samples were stored at −20 °C for future GC analysis.

### 3.8. Gas Chromatography

A Hewlett Packard 5890 series II model equipped with a flame ionization detector and non-polar capillary column (TG-SQC, 15 m, I.D.: 0.25 mm, Film: 0.25 μm) was used for the GC analysis. The initial temperature was set to 170 °C for 3 min and then ramped up at a rate of 20 °C/min to a final temperature of 280 °C, followed by an isothermal step for a total time of 30.5 min. The inlet temperature was 245 °C and the detector temperature was set to 280 °C. Cholesterol was used as a control to demonstrate retention time standards. The sterol profile of the samples isolated after UV exposure was analyzed after injecting 1 µL of sample into the GC, then plotting retention time against detector intensity. The peaks in the samples were analyzed using retention time in relation to the standard cholesterol retention time. The concentration of the cholesterol in each sample was calculated by the ratio of the area of the standard cholesterol peak compared to the cholesterol peak area of each sample.

## 4. Conclusions

Exposure of CHO-K1 cells to ZnO nanoparticles and UV-C irradiation proved to be damaging in terms of cell viability. UV-visible spectroscopy revealed that the 250 µg/mL of ZnO dose corresponds to the maximum absorption of ZnO nanoparticles. Similarities in cell morphology were observed when CHO cells were exposed to either UV radiation or ZnO nanoparticles. However, agarose gel electrophoresis performed after treatment demonstrated possible depletion in the ability to produce RNA after the cells were treated with ZnO concentrations of 25 µg/mL and higher, which suggests that the principal mode of toxic action of ZnO is through the generation of reactive oxidative species. Gas chromatography traces revealed that CHO-K1 irradiated with UV-C for 0, 60, 240, and 360 min had dramatic increases in cholesterol concentration present in cellular membranes compared to the wild-type CHO cells that received no UV treatment. The sharpest increase in cholesterol was observed at 360 min of exposure, which corresponded to about 20% cell viability.

## Figures and Tables

**Table 1:** The GC chromatogram areas, concentration (µg/µL and µM), percent viability, and calculated corrected cholesterol concentration (µg/µL and µM) based on cell viability after UV exposure starting with 1 × 10^7^ cells per sample ^a^.

UV Exposure Sample (min)	Area (10^5^) (a.u.)	Calc. (µg/µL)	Calc. µM (10^−2^)	Viability (%)	Corrected (µg/µL)	Corrected µM (10^−2^)
Standard	2.34	1.00	1.00	-	-	-
0	8.76	3.74	0.97	100.0	3.74	0.97
60	6.48	2.77	0.72	78.1	3.55	0.92
240	12.37	5.29	1.37	36.3	14.58	3.77
360	37.79	16.17	4.18	20.0	80.83	20.90

^a^ UV exposure sterol samples were dissolved in 20 µL of methanol prior to GC injection.

**Table 2:** The concentration and volumes of ZnO and the culture medium used for ZnO treatment in a 12-well plate ^a^.

Flask	[ZnO] (µg/mL)	[ZnO] (µM)	Cell + Media (µL)	ZnO + Media (1 mg/mL) (µL)	Media (mL)	Total (mL)
1	0	0.0	650	0	4.350	5
2	5	61.5	650	25	4.325	5
3	10	123.0	650	50	4.300	5
4	15	184.5	650	75	4.275	5
5	25	307.5	650	125	4.225	5
6	50	615.0	650	250	4.100	5
7	100	1230.0	650	500	3.850	5

^a^ CHO cells were seeded at 5 × 10^6^ cells/well.

**Table 3:** The concentration and volumes of ZnO and the culture medium used for ZnO treatment in a T-25 culture flask ^a^.

Sample	[ZnO] (µg/mL)	[ZnO] (µM)	Cell + Media (µL)	ZnO + Media (0.1 mg/mL) (µL)	Media (µL)	Total (µL)
1	0	0.0	100	0	900	1000
2	5	61.5	100	50	850	1000
3	10	123.0	100	100	800	1000
4	15	184.5	100	150	750	1000
5	20	246.0	100	200	700	1000
6	25	307.5	100	250	650	1000
7	50	615.0	100	500	400	1000
8	100	1230.0	100	100 *	800	1000
9	250	3075.0	100	250 *	650	1000
10	500	6150.0	100	500 *	400	1000

^a^ CHO cells were seeded at 5 × 10^6^ cells/flask. * Taken directly from 1.0 mg/mL stock of ZnO/Ethanol.
